# Memory Maintenance in Synapses with Calcium-Based Plasticity in the Presence of Background Activity

**DOI:** 10.1371/journal.pcbi.1003834

**Published:** 2014-10-02

**Authors:** David Higgins, Michael Graupner, Nicolas Brunel

**Affiliations:** 1 IBENS, École Normale Supérieure, Paris, France; 2 Departments of Statistics and Neurobiology, University of Chicago, Chicago, Illinois, United States of America; 3 Center for Neural Science, New York University, New York, New York, United States of America; UCL, United Kingdom

## Abstract

Most models of learning and memory assume that memories are maintained in neuronal circuits by persistent synaptic modifications induced by specific patterns of pre- and postsynaptic activity. For this scenario to be viable, synaptic modifications must survive the ubiquitous ongoing activity present in neural circuits *in vivo*. In this paper, we investigate the time scales of memory maintenance in a calcium-based synaptic plasticity model that has been shown recently to be able to fit different experimental data-sets from hippocampal and neocortical preparations. We find that in the presence of background activity on the order of 1 Hz parameters that fit pyramidal layer 5 neocortical data lead to a very fast decay of synaptic efficacy, with time scales of minutes. We then identify two ways in which this memory time scale can be extended: (i) the extracellular calcium concentration in the experiments used to fit the model are larger than estimated concentrations *in vivo*. Lowering extracellular calcium concentration to *in vivo* levels leads to an increase in memory time scales of several orders of magnitude; (ii) adding a bistability mechanism so that each synapse has two stable states at sufficiently low background activity leads to a further boost in memory time scale, since memory decay is no longer described by an exponential decay from an initial state, but by an escape from a potential well. We argue that both features are expected to be present in synapses *in vivo*. These results are obtained first in a single synapse connecting two independent Poisson neurons, and then in simulations of a large network of excitatory and inhibitory integrate-and-fire neurons. Our results emphasise the need for studying plasticity at physiological extracellular calcium concentration, and highlight the role of synaptic bi- or multistability in the stability of learned synaptic structures.

## Author Summary

Synaptic plasticity is widely believed to be the main mechanism underlying learning and memory. In recent years, several mathematical plasticity rules have been shown to fit satisfactorily a wide range of experimental data in hippocampal and neocortical *in vitro* preparations. In particular, a model in which plasticity is driven by the postsynaptic calcium concentration was shown to reproduce successfully how synaptic changes depend on spike timing, specific spike patterns, and firing rate. The advantage of calcium-based rules is the possibility of predicting how changes in extracellular concentrations will affect plasticity. This is particularly significant in the view that *in vitro* studies are typically done at higher concentrations than the ones measured *in vivo*. Using such a rule, with parameters fitting *in vitro* data, we explore how long the memory of a particular synaptic change can be maintained in the presence of background neuronal activity, ubiquitously observed in cortex. We find that the memory time scales increase by several orders of magnitude when calcium concentrations are lowered from typical *in vitro* experiments to *in vivo*. Furthermore, we find that synaptic bistability further extends the memory time scale, and estimate that synaptic changes *in vivo* could be stable on the scale of weeks to months.

## Introduction

Many experiments have shown that long-lasting changes in synaptic efficacy can be induced by spiking activity of pre- and postsynaptic neurons [1], [2]. In hippocampal and neocortical *in-vitro* preparations, both long-term potentiation and depression can be induced by protocols in which pre- and postsynaptic neurons emit tens to hundreds of spikes in specific temporal patterns [3]–[10]. In those preparations, plasticity has been shown to depend both on relative timing of pre- and postsynaptic spikes (‘spike timing dependent plasticity’, or STDP), and the firing rates of pre- and postsynaptic neurons. These induced changes in the connectivity of a neural circuit have then been assumed to maintain or initiate a long-term memory trace of external inputs that triggered these synaptic changes [11]. However, in order for this theory to be valid, the induced synaptic changes need to survive both activity triggered by other inputs, and the ongoing background activity that is pervasive in cortex [12], [13]. How changes in synaptic connectivity survive the continuous presentation of other inputs has been the subject of several studies [14], [15]. Here, we study the decay of the synaptic memory trace due to background activity using a theoretical approach.

Synaptic plasticity has been described using a multitude of different models and approaches [6], [7], . In early plasticity models, synaptic changes were purely induced by the firing-rates of pre- and postsynaptic neurons [16], [17], [19]. At the end of the nineties, theorists introduced purely spike-timing based models [21], [23]. Finally, more recent models have been striving to capture a wide range of experimental data, and as a result capture both the spike-timing and firing rate dependence of synaptic plasticity [6], [24]–[31]. These models are natural candidates for studies of the stability of synaptic changes during ongoing activity. In this paper, we choose the model of Graupner and Brunel [28] for the following reasons: (i) the model includes the calcium concentration in the post-synaptic spine, which is known to be a crucial link between pre- and postsynaptic activity and long-term synaptic changes; (ii) the model exhibits bistability of synaptic changes accounting for experimental evidence suggesting that CA3-CA1 synapses in the hippocampus are bistable [32], [33]; (iii) the model is simple enough that the dynamics of the synaptic efficacy variable can be computed analytically.

Postsynaptic calcium entry has been identified to be a necessary [34]–[36] and sufficient [37]–[39] signal for the induction of synaptic plasticity (but see ref. [40]). However, most of the *in vitro* experiments evoking synaptic changes use elevated extracellular calcium concentrations, while *in vivo* calcium levels are estimated to be around 1.5 mM [41]. The impact of reduced calcium entry due to the lower extracellular calcium concentration *in vivo* on the time scale of synaptic decay has not been considered heretofore.

In the present paper, we study the persistence of synaptic changes, first in a synapse connecting a pair of independent Poisson neurons, and second in a large network of excitatory and inhibitory leaky integrate-and-fire (LIF) neurons. We show that in the absence of bistability, synaptic changes decay exponentially during ongoing activity and that the time constant exhibits a power-law like behaviour with respect to the present firing rate. We demonstrate that the reduced extracellular calcium concentration *in vivo* leads to several orders of magnitude longer memory time scales. The introduction of bistability in the synaptic plasticity rule further stabilises synaptic changes at low firing rates and extensively prolongs memory time scales when combined with the *in vivo* extracellular calcium conditions. Finally, we extend our results to a large recurrent network of LIF neurons, where we demonstrate network firing rate stability under synaptic plasticity, decay of an implanted memory for *in vitro* parameters and long term memory maintenance for *in vivo* parameters.

## Results

Memories are thought to be stored in the brain thanks to activity-dependent modifications of synaptic connectivity. According to this hypothesis, memories stored by a particular neural circuit are encoded by the state of all the modifiable synapses of the circuit. Synaptic plasticity allows particular patterns of activity to leave a trace in the connectivity matrix, but this trace is then potentially vulnerable to the ongoing activity that follows. An important question is therefore what controls the time scale of the persistence of a particular synaptic state, in the presence of such ongoing activity. To study this question, we initialize the efficacy of a synapse (either an isolated one, or part of a network) at a particular value, and study how this efficacy decays with time in the presence of background activity, using a calcium-based model of synaptic plasticity [28].

In the model, the temporal evolution of the synaptic efficacy variable, 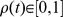, is described by 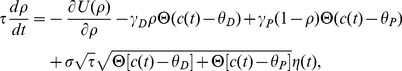
(1)where 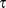 is the time constant of synaptic efficacy changes, and 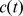 is the calcium concentration. The dynamics of 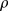 depends on four terms:

1. The dynamics are governed by a potential 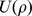 for low calcium concentrations (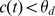) since all other terms on the right-hand side of Eq. (1) are zero then. In the following we consider two possible scenarios for 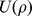: (i) a flat potential, 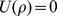 - in this case the synaptic efficacy variable stays constant in time in the absence of calcium transients. This means all possible values of 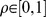 are stable; (ii) a double well potential, 
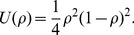
(2)


In this case, 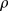 evolves towards one of two possible stable fixed points (the minima of 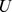), one at 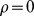 - the DOWN state -, the other at 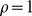 - the UP state -, depending on the initial condition.

This corresponds to a bistable synapse.

2. For intermediate calcium concentrations (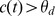), the synapse is depressed, with a depression rate 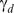.

3. For large calcium concentrations (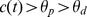), the synapse undergoes both potentiation, with a potentiation rate 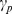, and depression, with the same rate as in the 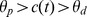. Since 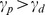, potentiation dominates over depression in that region.

4. A noise is only active when calcium concentration is above the lowest plasticity threshold 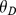, and increases in magnitude when the upper plasticity threshold, 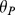, is also crossed. 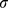 defines the amplitude of the noise, and 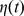 is a Gaussian white noise process with unit variance.

Changes in 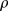 are induced by increases in the postsynaptic calcium concentration, 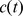 (see Eq. (11), in Methods), evoked by pre- and postsynaptic spikes. The calcium concentration increases by an amount 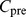, in response to presynaptic spikes, while it increases by an amount 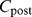 in response to postsynaptic spikes. It decays exponentially with a time constant 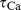 in between spikes. Calcium transients induced by presynaptic activity are assumed to represent calcium influx through NMDA receptors, while calcium transients induced by postsynaptic spikes are assumed to represent activation of voltage-gated calcium channels [42] (see [28] for more details of the model).

This calcium-based model of synaptic plasticity has been used to successfully fit data from various experimental preparations [28]. Here, we use the data-set that best fits plasticity data obtained in visual cortex slices [6] - hereafter called the ‘*in vitro*’ parameter set. In this experiment, the extracellular calcium concentration was set to be 2.5 mM [6], which is significantly higher than the estimated *in vivo* concentration of about 1.5 mM [41]. Here we assume that a decrease in extracellular calcium concentration leads to a proportional decrease in the calcium influx into the post-synaptic spine. Using this assumption, we can readily predict the effects of decreasing the extracellular calcium concentration on the plasticity rule in the calcium-based model by scaling the amplitudes of the pre- and postsynaptically evoked calcium transients according to the ratio of calcium concentrations, i.e. 1.5/2.5 = 0.6. This leads to what we call the ‘*in vivo*’ parameter set. Values of all parameters for both conditions are indicated in Table 1.

**Table 1:** Parameters of the calcium-based synapse model.

Parameter	In-vitro	In-vivo
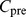	0.56175	0.33705
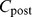	1.23964	0.74378
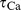 (ms)	22.6936	
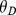	1	
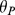	1.3	
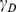	331.909	
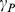	725.085	
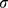	3.3501	
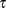 (sec)	346.3615	
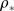	0.5	
D (ms)	4.6098	

The *in vitro* values are obtained in [28] by fitting the model, using a gradient descent method, to cortical plasticity data presented in Figure 8a (frequency dependence of synaptic plasticity for a fixed relative pre- post- spike timing) of [6]. *In vivo* calcium amplitudes are scaled from the *in vitro* values according to the change in extracellular calcium concentration.

**Figure 1:**
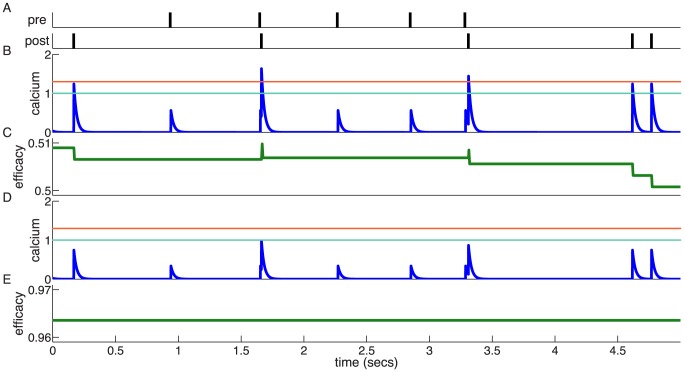
Dynamics of the synaptic plasticity model with the *in vitro* and *in vivo* parameter sets. (**A**) Pre- and postsynaptic spike trains generated as realisations of Poisson processes at 1/s. (**B,C**) The spike train in *A* induces large calcium transients (blue trace) with the *in vitro* parameter set (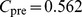 and 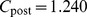; see Table 1). Whenever the calcium trace crosses the depression (cyan) or potentiation thresholds (orange), changes in the synaptic efficacy (green) are induced. (**D,E**) Same as in *B,C* but with calcium amplitudes corresponding to the *in vivo* case (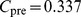 and 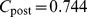). The small calcium transients do not cross the depression/potentiation thresholds and no efficacy changes are observed. Note that the flat potential for 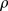 is used here and that noise is set to zero for clarity, 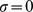.

The dynamics of the synaptic efficacy in response to calcium transients under the *in vitro* and the *in vivo* conditions are illustrated in Fig. 1. The synaptic efficacy is only modified when the calcium concentration increases above the depression threshold 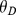 (Fig. 1,**B–E**). For the *in vitro* case, this happens whenever a postsynaptic spike occurs since 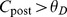, but for the *in vivo* parameter set this happens much more rarely because of the smaller calcium amplitudes (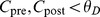 in the *in vivo* case; see Table 1). In the latter case, synaptic changes are only induced whenever subsequent spikes occur in short succession such that calcium accumulates and crosses the depression and/or potentiation threshold. Such events are rare at low firing rates (Fig. 1,**D–E**).

### Memory decay for a synapse connecting two independent Poisson neurons

We now proceed to study the time scales of synaptic decay. We start with the case of a synapse connecting two neurons firing according to uncorrelated Poisson processes, and compare the memory time constants in the flat and double-well potential cases. Simulations were performed using an event-based implementation of the synaptic plasticity model, which updates the synaptic efficacy only upon the occurrence of pre- and postsynaptic spikes (see Methods for details).

**Figure 2:**
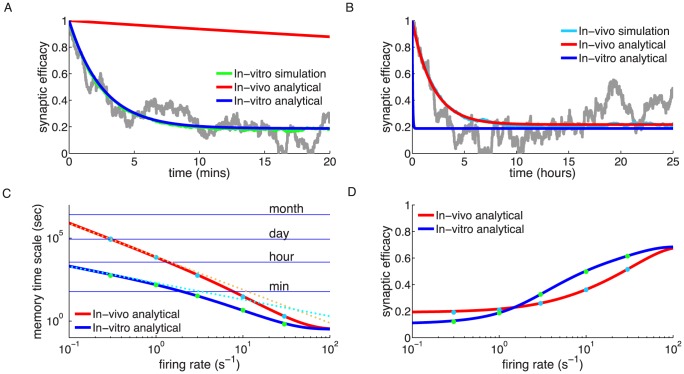
Memory decay for a single synapse with flat potential in the presence of uncorrelated pre- and postsynaptic Poisson firing. **(A,B)** Temporal evolution of the mean synaptic efficacy in the presence of pre- and postsynaptic Poisson firing at 1/s for the *in vitro* (green in *A*) and the *in vivo* (light blue in *B*) parameter sets (mean shown for 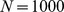 synapses). Blue and red lines show the mean dynamics as predicted by the Ornstein-Uhlenbeck theory. Grey lines show example traces of synaptic efficacy evolution in time. **(C)** Decay time constant as a function of the firing rate for *in vitro* and *in vivo* parameter sets. The blue and red lines show the calculated decay time constant, 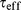, from the OU theory. The points show exponential decay times obtained by fitting single exponential decay functions to the mean synaptic dynamics as shown in *A* and *B* illustrating that the OU theory correctly describes the full model behaviour. The cyan and orange dotted lines illustrate the derived power law behaviour, 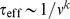, between memory time scales and low firing rates (see text). The power reflects the number of spikes required to cross the plasticity thresholds, that is, 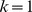 for *in vitro* (cyan dotted line) and 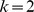 (orange dotted line) for *in vivo* case. **(D)** Asymptotic synaptic efficacy as a function of the firing rate for *in vitro* and *in vivo* parameter sets. The lines show the calculated asymptotic value, 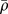, from the truncated OU theory (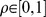) for *in vitro* (blue line) and *in vivo* (red line) cases. Note that at high frequencies 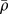 saturates at a value equal to 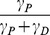, since both depression and potentiation terms are active in the high calcium region. The points show steady-state values obtained by fitting single exponential decay functions to the mean synaptic dynamics as shown in *A* and *B* (green: *in vitro*; light blue: *in vivo*).

We initialise the synaptic efficacy to 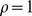 and investigate the time constant of decay in the presence of an ongoing constant firing rate, initially for the flat potential synapse (Eq. 1, with 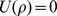). Pre- and postsynaptic neurons emit uncorrelated spikes following Poisson statistics, both with a mean rate of 1/s. Under these conditions, a fully potentiated synapse progressively decays and eventually fluctuates around a value of 0.2. On average, this decay is well described by a single exponential function (Fig. 2,**A,B**). The time constant of this decay is much longer in the case of the *in vivo* parameter set (Fig. 2,**B**) than in the *in vitro* parameter set (Fig. 2,**A**). The decay time constant is 2.5 minutes for the *in vitro* case and approximately 2 hours for *in vivo* in the presence of 1/s pre- and postsynaptic firing (Fig. 2,**C**).

The dynamics of the synaptic efficacy (Eq. 1) can be described by a truncated Ornstein-Uhlenbeck (OU) process if single calcium transients induce small changes in the synaptic efficacy and if the potential is flat (see [28] for the non-truncated case). Truncation of the process is induced by the bounds at 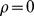 and 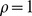. In such a process, the mean synaptic efficacy decays exponentially with a time constant, 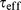, which is given by 
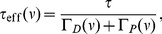
(3)to an asymptotic average efficacy, 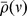 (see Eq. (22) in Methods), where 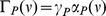 and 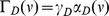 are the net potentiation and depression rates which depend on the rates 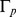 and 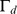 as well as on the average fractions of time spent above the potentiation and depression thresholds, 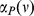 and 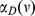, respectively. The average fractions of time the calcium traces spend above the potentiation and depression thresholds are given by



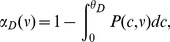
(4)

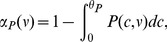
(5)where 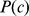 is the probability density function of the calcium variable, that can be computed analytically in the case of independent pre- and postsynaptic Poisson firing [28], [43] (see Methods for details). The theory provides an excellent match for the dynamics of the mean synaptic efficacy – compare in Fig. 2,**A,B** the truncated OU theory (blue and red curves), with the simulation mean (green and light blue curves).

Synaptic efficacy decay becomes faster with increasing pre- and postsynaptic firing rates since the calcium trace spends more time above depression and potentiation thresholds (Fig. 2,**C**). At the same time, the asymptotic value of synaptic efficacy (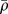) increases due to an increase in time spent above the potentiation threshold (Fig. 2,**D**). As a result of the smaller *in vivo* calcium amplitudes, the efficacy decay for the *in vivo* case is, at all firing rates, much slower than the decay *in vitro* (Fig. 2,**C**). The asymptotic efficacy value is lower, at small firing rates (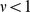/s), for the *in vitro* case since isolated postsynaptic spikes always cross the depression threshold (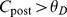) which results in a large net depression rate 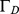, compared to *in vivo* (Fig. 2,**D**).

To get a deeper understanding of the dependence of the memory time scale on the firing rates of pre- and postsynaptic neurons, we set 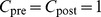. This simplification allows us to derive a power law relationship between the memory time scale and the firing rate 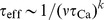, where 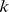 is the number of (pre and/or post-synaptic) spikes required to clear the depression/potentiation thresholds. To compute the memory time scale, we need to compute the fraction of times spent above the depression and potentiation thresholds, 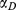 and 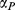. In the case 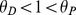, one can show that at low rates 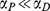. Consequently it is only necessary to focus our analysis on 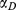. When 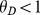, the time spent above the depression threshold is 
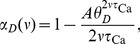
(6)where 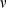 is the firing rate of pre- and postsynaptic neurons (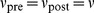), 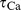 is the decay constant for the calcium concentration and 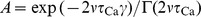 (see Eqs. (13) – (17)). This closed form solution allows us to perform an expansion for low firing rates 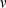




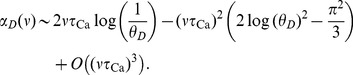
(7)Similarly for 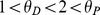 we have in the low rate limit, 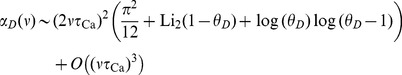
(8)where 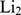 is the dilogarithm, 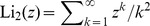. Thus, in both cases we find that the memory time scale depends on the firing rate as
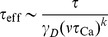
(9)where 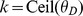. We expect this relationship to hold in general. Intuitively, this is due to the fact that we need 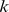 spikes arriving simultaneously on a time scale of order 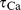 in order for the calcium concentration to cross the depression threshold 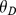, and that the probability of observing 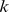 spikes in a time interval 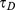 is at low rates proportional to 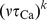. We also expect the result to hold in general for 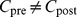. In this case, we expect that 
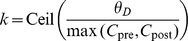
.

The derived power law behaviour for 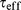 is plotted in Fig. 2,**C** together with the full analytical solution for 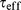. We see that as expected, 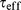 scales as 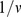 for the *in vitro* parameter set, where a single spike is enough to cross 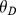, while it scales as 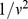 for the *in vivo* parameter set, where two spikes are needed to cross the depression threshold.

The implication of this theoretical result is that, at low firing rates, there is a direct relationship between the number of spikes required to clear the lower plasticity threshold and the memory time scale. Note that the full synaptic efficacy model with 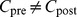 is considered in the following (see Table 1)

### Memory decay for a bistable synapse

We now turn to examine the effect of a bistability on memory time scales. The dynamics of the synapse is now described by Eq. (1), where the potential 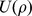 is given by Eq. (2). This double well potential leads to a bistable synapse, that can take two possible efficacy states (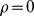 and 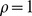) in the absence of activity. In the presence of background activity, transitions between these two states become possible. We investigate stability and transition times for *in vitro* and *in vivo* parameter sets as a function of pre- and postsynaptic firing rates.

The effect of background activity on the dynamics of 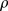 can be explained by the fact that it modifies the potential, 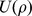, leading to an *effective potential*

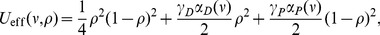
(10)


(see Methods). In Eq. (10), the first term on the r.h.s. corresponds to the ‘bare’ double well potential 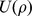 (Eq. (2)); the second term describes the effect of depression on the potential, that tends to strengthen the stability of the lower well (DOWN state) at 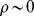, at the expense of the other well that tends to disappear when 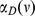 increases; finally, the last term describes the effect of potentiation, that shifts the minimum of the only remaining well towards higher values of 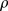 when 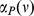 increases.

**Figure 3:**
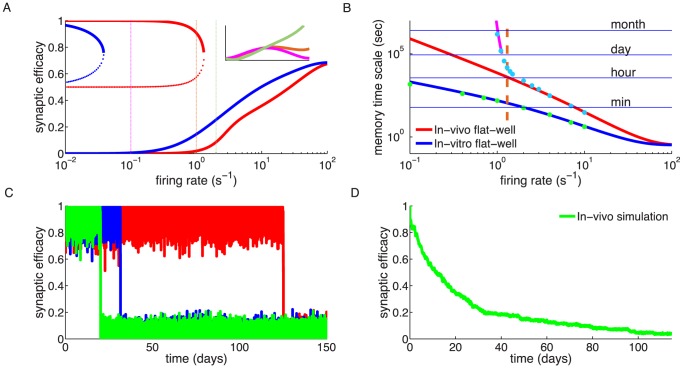
Memory decay for a bistable synapse in the presence of uncorrelated pre- and postsynaptic Poisson firing. (**A**) Steady-states of synaptic efficacy as a function of firing rate for the *in vitro* (blue) and the *in vivo* (red) parameter sets. Stable states are shown by solid lines and unstable states by dotted lines. Synaptic efficacy is bistable at low rates (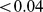/s for *in vitro* and 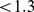/s for *in vivo*) and monostable at high firing rats. The effective potential of synaptic efficacy is shown for three firing rates (0.1/s - magenta line; 1/s - orange line; 2/s - green line) and the *in vivo* parameter set in the inset (firing rates indicated by vertical lines). (**B**) Decay time constant as a function of the firing rate for the *in vitro* and the *in vivo* parameter sets. For the *in vivo* parameter set below 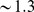/s, the bistability greatly extends memory time scale compared to a synapse with flat potential (red line) and can be predicted using Kramers escape rate (magenta line). The vertical dashed line illustrates the frequency at the *in vivo* bifurcation point. For the *in vitro* parameter set, the bistability has no influence on decay time constants for firing rates above 0.1/s. The points show exponential decay times obtained by fitting single exponential decay functions to the mean synaptic dynamics. (**C**) Individual synaptic efficacy traces for the *in vivo* parameter set at 1/s pre- and postsynaptic firing. The synapses remain in the upper potential well for a long time and stochastically cross the potential barrier to the low efficacy state. (**D**) Averaged synaptic efficacy trace of many synapses for the *in-vivo* parameter set at 1/s. The bistability extends the memory time scale from hours for a flat potential to days.

Thus, there are two distinct regions of firing rates in the bistable case with respect to the effective potential. For sufficiently low rates, the effective potential still has two minima (see Fig. 3,**A**, and the effective potentials for 0.1/s and 1/s, indicated by orange and magenta curves in the inset). There is a critical value of the rates at which the high efficacy minimum disappears through a saddle-node bifurcation. Beyond this rate, the synapse is no longer bistable, and synaptic efficacy has one stable state only (Fig. 3,**A**), equivalent to the asymptotic efficacy value for the flat potential (Fig. 2,**D**). Finally, at high firing rates, the ‘bare’ potential becomes negligible, and the effective potential approaches a quadratic potential with a single stable state whose location depends on the rate (green curve in the inset in Fig. 3,**A**). The transition from double-well to single well regimes occurs at different firing rates for the *in vitro* (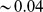/s) and the *in vivo* (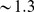/s) parameter sets due to the larger calcium amplitudes in the former.

For the *in vitro* parameter set, adding bistability to the synaptic efficacy has no influence on the decay time constant for firing rates larger than approximately 0.1/s (Fig. 3,**B**). In contrast, for the *in vivo* parameter set, bistability considerably prolongs memory decay times with respect to synapses with flat potential at firing rates below <1.4/s. In the presence of two stable states, the decay of memory occurs only due to synaptic noise fluctuations that push the synaptic efficacy out of the upper well. The influence of the double well potential on the dynamics of the synaptic efficacy traps synapses in the UP state leading to long dwell times before crossing the potential barrier and converging to the low efficacy state (Fig. 3,**C**). The double-well has a prolongation effect on memory duration up to firing rates of about 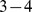/s due to the transition between double-well and single-well regimes. At high firing rates, the potentiation and depression processes dominate and the effects of the double-well becomes negligible for both parameter sets, that is, the decay time constant is indistinguishable between flat and double-well potential synapses (see Fig. 3,**B**).

For low firing rates, we can accurately predict the increase in the decay time constant in the presence of bistability using Kramers escape rate for the mean first passage time across a potential barrier (Fig. 3,**B**; see Methods Eq. (26)). In this regime, we calculated an effective decay time constant using Kramer's escape theory given by 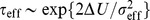, where 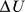 is the height of the effective potential barrier and the noise term, 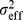, drives the escape of the efficacy from the upper stable state (see magenta line in Fig. 3,**B** for the *in vivo* case). Both terms 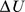 and 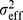 are dependent on 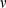 and are detailed, along with 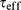, in Eqs. (23) and (26) (see Methods for more details). In the low rate limit, 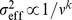 and therefore the memory time scale increases exponentially with the inverse of the rate to a power 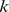, 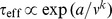, where 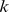 is again the number of simultaneous spikes needed to cross the depression threshold. This exponential dependence extends the time scale for synaptic decay at 1/s to the order of one month for a bistable synapse with the *in vivo* parameter set, up from hours for a synapse with flat potential.

### Steady-state behaviour of networks of LIF neurons with plastic synapses

We next study the behaviour of the calcium-based synaptic plasticity model in a recurrent network of spiking neurons. We first examine the steady-state of synaptic efficacy and network activity. We again make use of the event-based implementation of the synaptic plasticity rule (described in Methods) allowing us to simulate much longer time scales than are normally attainable by a time stepping simulator.

The recurrent network consists of 8000 excitatory and 2000 inhibitory leaky integrate-and-fire (LIF) neurons. Each neuron receives an external input which consists of a constant (DC) term and a white noise term. External noise is independent from neuron to neuron. Each neuron also receives synaptic inputs from other neurons in the network. The connection probability between any two neurons is 0.05 and uniform in space and across neuron types. Synapses between excitatory neurons are plastic according to the calcium-based plasticity model (Eq. 1), while all synapses involving inhibitory neurons are fixed. Parameters of the network are chosen so that the network settles in a stable asynchronous irregular state [44]. Hence, correlations between neurons are weak. See Methods for more details of the network model.

**Figure 4:**
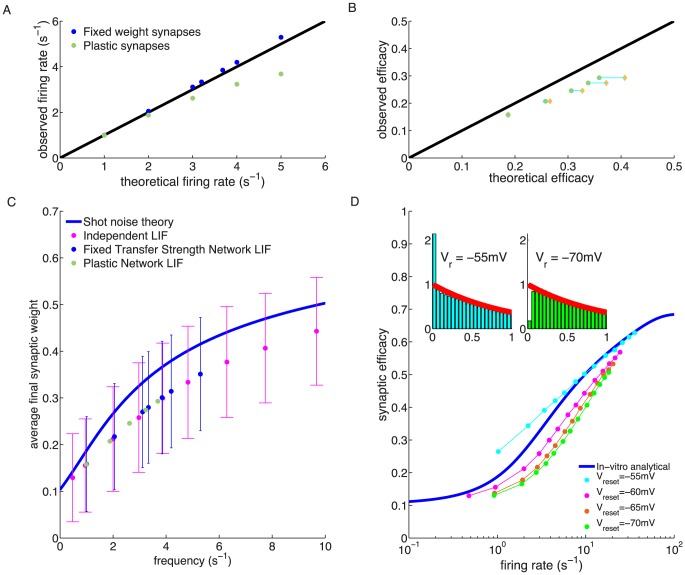
Steady-state behaviour of a recurrent network with plastic synapses between excitatory neurons. (**A**) Firing rate mean-field predictions compared with network simulation results. The mean-field theory predicted firing rate is higher (black line) than the actual firing rate of the excitatory neurons (green dots) in the recurrent network of 8000 exc. and 2000 inh. LIF neurons. Network simulation with fixed synapses yield a good match with the theory (blue dots). (**B**) Average synaptic weight prediction compared with asymptotic average synaptic weights in the network simulation. The observed average synaptic efficacy of excitatory to excitatory connections is smaller (mustard dots) than the theoretical prediction (black line). Even when using the asymptotic firing rate of the network in the calculations (green dots), the average synaptic efficacy is overestimated by the theory. (**C**) Mean and standard deviation of synaptic weights vs. firing rate for independent LIF neurons (magenta), networked LIF neurons (green) and LIF neurons in a network in which actual weights are held constant but we examine how their efficacy would have evolved in the presence of observed firing (blue dots). Asymptotic synaptic weights for LIF neurons underestimate the efficacy predicted by the theory (blue line). (**D**) Average synaptic weight vs. firing rate for independent LIFs with different reset potentials. The analytical prediction of the asymptotic synaptic weight based on Poisson firing is shown by the blue line (same as in *C*). The reset potential in the LIF model, 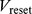, has a marked influence on the observed average synaptic efficacy. Depolarised/hyperpolarised reset potentials (e.g. −55/−70 mV, cyan/green dots) lead to an over/under-representation of short ISIs (left/right inset) compared to Poisson neurons (red line in insets). ISI histograms in inset are shown for LIF neurons firing at 1/s.

The fixed point of the network can be determined analytically by solving a set of three self-consistent equations for the excitatory and inhibitory mean rates as well as for the mean excitatory-to-excitatory (E
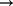
E) synaptic efficacy (see Methods). Two of these equations give the stationary firing rates of excitatory and inhibitory populations (Eqs. (32) – (33)), as a function of the mean E
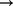
E synaptic efficacy [44], [45]. The third equation gives the mean E
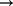
E synaptic efficacy as a function of the firing rate of the excitatory population, assuming Poisson firing statistics of neurons (Eq. (22)). Starting from the analytically determined initial conditions, the recurrent network converges to a steady-state of constant average firing rates of all neurons in the network, and constant average synaptic efficacy of the plastic connections. Figure 4,**A** shows how the firing rates observed in the simulations compare with the analytically predicted firing rates. It shows that at sufficiently low rates, the analytical prediction gives a very good estimate of the observed rates; however, for rates above 3Hz the observed rates are significantly lower than the analytical prediction. Likewise, the analytical prediction for the mean E
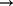
E synaptic efficacy significantly overestimates the observed efficacies (green dots in 4,**B**).

What is the source of the difference between theory and simulations in predicting the steady network state? When synapses are fixed in the network at the efficacies predicted by the corresponding firing rate, the analytically predicted network firing rates provide a good approximation of the observed activity (blue dots in 4,**A**). This suggests that the underestimation of firing rates and synaptic efficacy emerges from the mapping of firing rates onto synaptic efficacy, and not due to correlations between spike trains of different neurons. We examine this effect in Fig.4,**C**, where we show asymptotic mean synaptic efficacy results under three different conditions. First, we simulated a population of disconnected, independent LIF neurons, receiving stochastic independent inputs with the same mean and variance as in the ‘real’ network simulation (magenta dots). By definition, this simulation led to uncorrelated spike trains. In this simulation, fake synaptic connections between neurons obeyed the plasticity rule, but had no effect on the dynamics of the neurons. Second, we simulated a connected recurrent network with constant synaptic weights. As in the first case, we simulated ‘fake’ synaptic connections that obeyed the standard plasticity rule, but these fake synapses had no effect on the dynamics (blue dots). Third, we simulated a standard recurrent network in which synaptic weights are plastic according to the plasticity rule (green dots). All three simulations show indistinguishable results, and in all three cases the average (real or fake) synaptic efficacies are consistently smaller compared to the analytical shot noise prediction (4,**C**, blue line). This suggests that correlations have a negligible effect on mean efficacies and firing rates, and that the differences between simulations and theory are due to differences in spiking statistics between the LIF model and a Poisson process.

To investigate further how the spiking statistics of the LIF model and in particular the interspike-interval (ISI) distribution causes the differences seen in Fig. 4, we varied the ISI distribution of the LIF neuron by changing the reset potential (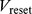, see Methods). This change had a strong effect on the average synaptic efficacy (4,**D**). A reset potential close to threshold (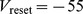 mV, 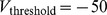 mV) yields an overrepresentation of short ISI compared to Poisson firing (4,**D**, inset) and in turn overestimates the average synaptic efficacy (4,**D**; cyan dots). Conversely, more depolarised reset potentials lead to an under-representation of short ISIs with regard to Poisson firing and consequently to an underestimation of the average synaptic efficacy (4,**D**; magenta, red and green dots). We use an intermediate value of 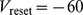 mV in the following network investigations.

To conclude this section, the calcium-based synaptic plasticity rule does not affect the stability of the asynchronous irregular state in a large recurrent network of LIF neurons. Since LIF neurons in the network exhibit ISI distributions which deviate from those of Poisson neurons, the accuracy of our calculation of the average synaptic efficacy which is based on Poisson firing decreases with increasing firing rates up to a certain point. At high firing rate, calcium remains above the plasticity thresholds most of the time and the fraction of time spent above the thresholds converges to one, irrespective of the underlying neuron model.

### Memory decay in a recurrent network of LIF neurons

**Figure 5:**
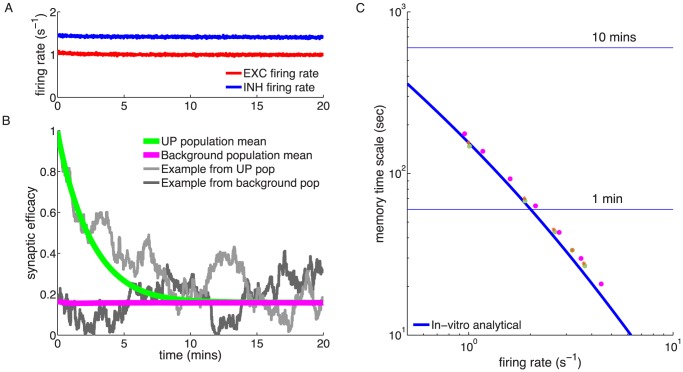
Memory decay for a subset of potentiated synapses in a recurrent network with the *in vitro* parameter set. (**A**) Temporal evolution of the average excitatory (red) and inhibitory (blue) firing rate. A network of 10,000 LIF neurons is initialised at the theoretically predicted steady-state and simulated for 20 min real time. **(B**) Temporal dynamics of synaptic efficacies in the network. The majority of synapses are initialised to the theoretically predicted asymptotic synaptic efficacy (mean: magenta; single synapse example: dark gray). A randomly selected subset of 5% are set to 1 at the beginning of the simulation (mean: green; single synapse example: light gray). (**C**) The exponential decay time constant of the potentiated synapses. The value obtained from fitting a single exponential to the mean decay (green dots) is well approximated by the analytically calculated decay time constant from the OU process (Eq. (3)). Introduction of a double-well potential does not modify the memory time constant for the *in vitro* parameter set (orange stars). The slight deviation of the decay time constants with respect to the OU theory, that is, the network decay time constants are slower, are due to the LIF firing statistics as can be seen from the comparison with independent LIF neurons (magenta dots).

Finally, we examine the decay of a memory trace in a network for the *in vitro* and the *in vivo* parameter set. We initialise all excitatory-to-excitatory synaptic weights at their theoretically predicted asymptotic weights, except for a randomly selected subset of 5% which are set to a weight of 1. With the *in vitro* parameter set, the potentiated synapses decay relatively quickly to their asymptotic value (Fig. 5,**B**). The time course of the average decay can be described by a single exponential function and the decay time constant is well approximated by the time constant, 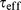, of synaptic decay from the truncated OU process (see Eq. (3); Fig. 5,**C**). This means that the average dynamics of synaptic decay in the network is equivalent to synapses driven by independent pre- and postsynaptic Poisson neurons firing at the same rate as the excitatory neurons in the network (compare to Fig. 3,**B**). The addition of the double-well potential does not change the decay time constant for the *in vitro* parameter set, as for a single synapse driven by independent pre- and postsynaptic Poisson firing (Fig. 5,**C** orange stars; compare with Fig. 3,**B**). The lack of short ISIs in LIFs compared to independent Poisson neurons leads to a small increase in observed decay times in the network as compared with the OU theory (see Fig. 5,**C**).

**Figure 6:**
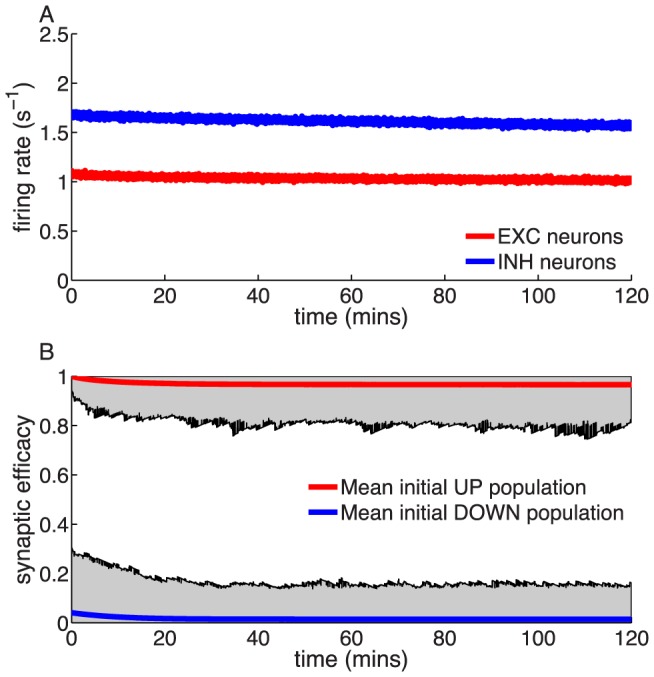
Memory decay for a subset of potentiated synapses in a recurrent network with the *in vivo* parameter set and double-well potential. **(A)** Temporal evolution of the average excitatory (red) and inhibitory (blue) firing rate. A network of 10,000 LIF neurons is initialised at the theoretically predicted steady-state and simulated for 120 min real time. **(B)** Temporal dynamics of synaptic efficacies in the network. The average dynamics of the 95% initialised in the DOWN state (blue) and the 5% initialised in the UP state (red) is shown. The shaded gray region represents the range of values visited by synapses in the UP and in the DOWN state populations, indicating that no transition occurs.

In contrast, when using the *in vivo* parameter set with the double-well potential, we observe that the potentiated synapses get locked in the UP state for the duration of the network simulation with an excitatory neuron firing rate of 1/s (Fig. 6,**C**). None of the synapses in the potentiated subset crosses the unstable fixed point and converges to the DOWN state during a network simulation of 120 min, neither does the reverse transition occur. We expect that the escape from the UP state will be predicted by Kramers escape rate (Eq. (26)) which correctly accounted for escape dynamics of an isolated synapes driven by independent pre- and postsynaptic Poisson processes (Fig. 3**B**). There, the decay time constant for a firing rate of 1/s is on the order of a month, a time scale that cannot be reached by our network simulation.

Hence, as in case of independent Poisson neurons, the combination of a double-well potential with the *in vivo* parameter set leads to several orders of magnitude longer memory time constants, compared to the *in vitro* parameter set and a flat potential.

## Discussion

In this paper, we studied the stability of synaptic efficacy, in a plastic synapse subjected to background activity of pre- and postsynaptic neurons. We used a calcium-based plasticity model that has been shown to fit experimental data in hippocampal and neocortical preparations [28]. The model was investigated numerically, using an event-based implementation of the plasticity rule, as well as analytically, using a diffusion approximation. Thanks to this formalism, we derived scaling laws that describe how memory time scale is related to the firing rates of pre- and postsynaptic neurons. At low firing rates, we find that, when synapses are monostable, synaptic efficacies decay to an equilibrium value with a time scale that depends on the firing rates as a power law, 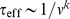, where *k* is the number of simultaneous spikes needed to cross the depression threshold. When synapses are bistable, memory decay is akin to diffusion of a particle out of a potential well, which leads to much stabler memories, with time scales that increase exponentially with the inverse of the firing rates, 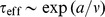, at low rates. We showed that these estimates accurately reproduce the results of simulations, both of a synapse connecting two isolated independent Poisson neurons, and of a large network of LIF neurons.

We have focused here on how changes in the amplitudes of the calcium transients affect memory time scales. A change in other model parameters also affects these time scales. Changing the depression threshold, for example, has a similar pronounced effect, since the exponent in the scaling law between memory time scale and background rate depends on the ratio between this threshold and the amplitudes of the calcium transients (see (9)). On the other hand, changing other parameters of the model (such as the time constants and the potentiation and depression rates) have much milder effects, in the flat potential regime, since the time scale depends algebraically on such parameters, rather than exponentially. In the absence of further data, we have assumed a linear relationship between the external calcium concentration and the calcium influx to explore the *in vivo* regime. A non-linear relationship between the extracellular calcium concentration and the concentration in postsynaptic microdomains - conjectured to be relevant for synaptic plasticity - should modify our results quantitatively rather than qualitatively.

Previous studies have investigated memory maintenance in networks of neurons connected by synapses endowed with standard spike-timing dependent plasticity rules [46]. Billings and van Rossum (2009) demonstrated that the memory time scale depends dramatically on whether the rule is additive or multiplicative. In a multiplicative STDP rule, in which synaptic change depends on the current value of the weight such that synaptic changes decrease when the weights approach the bounds, distributions of weights are unimodal [46]–[48] and the memory of synaptic changes decay as 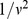, since synaptic changes occur upon coincidence of pre- and postsynaptic spikes in the characteristic time window of the STDP rule. These behaviours are very similar to the behaviour of the calcium-based rule in the flat potential case, in the parameter region in which two spikes are needed to cross the depression threshold. This is due to the fact that the calcium-based rule defined by Eq. (1) is multiplicative. In the calcium-based rule however, the exponent describing the memory decay at low rates can be set to an arbitrary integer number, through an appropriate rescaling of the ratio between the amplitude of the calcium transients and the depression threshold. In additive STDP rules, the picture changes dramatically and the synaptic weight distributions become bimodal, with weights attracted either to the lower or upper bounds through a symmetry breaking mechanism [23], [46]. In this situation, the memory time scales are much longer, and decay of synapses is similar to diffusion in a double well potential.

Several studies have shown that synaptic bi- or multi-stability can emerge from a number of mechanisms such as positive feedback loops in extensive protein signaling cascades [49], autophosphorylation of CaMKII [50]–[54], self-sustained regulation of translation [55], or modulation of receptor trafficking rates [56]. Such mechanims of bistablity are effectively implemented here in the form of the double well potential. Miller *et al.* (2005) studied the stability of the up state in a model of the bistable calcium/calmodulin-dependent protein kinase II system with respect to stochastic fluctuations induced by protein turnover [57]. They show that the CaMKII switch composed of a realistic number of CaMKII proteins is stable for years even in the presence of protein turnover, phosphatase as well as free calcium fluctuations. The transitions induced by background activity investigated here impose an upper limit on memory life-time which is typically lower, indicating that *in vivo* neuronal activity, not protein turnover, will be the limiting factor of memory life-times.

In vivo, memory in synaptic connectivity structures will be affected both by ongoing background activity, but also by changes in network activity induced by external stimuli. How ongoing presentations of external inputs affect memories of past stimuli has been the subject of several studies in recent years (e.g. [15], [20], [58]), in simpler networks of binary neurons. A detailed exploration of this issue in the model studied here is outside the scope of the present paper, but we anticipate that parameter regions that extend the robustness of synaptic memories in the face of background activity will also tend to protect the network against changes induced by external inputs.

Distributions of synaptic weights have been examined in a number of studies [4], [6], [59]–[62]. In all of these studies, distributions of synaptic weights appear unimodal and skewed, and peak at a low weight. In some cases, the distribution has been shown to be well fitted by a lognormal distribution [60], [62]. This seems at first sight at odds with the distributions of weights shown in the present paper, which are either a truncated Gaussian in the flat potential case, lacking the fatter tail of the lognormal distribution, or bimodal in the double-well case. However, the model in the flat potential case can be made consistent with the data, by choosing synaptic efficacy variables which are an exponential of the 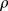 variable, rather than being linearly related to 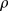. In this case, synaptic efficacies themselves become exponentiated Ornstein-Uhlenbeck processes, consistent with [62]. The model with a double-well potential could also be made consistent with a unimodal distribution, provided the synaptic up and down states are highly heterogeneous from synapse to synapse. Finally, we should point out that the distributions we observe are asymptotic distributions under a statistically constant distribution of inputs. Synapses in vivo are typically subjected to highly non-stationary firing rates of pre and post synaptic neurons. These non-stationarities can also potentially strongly affect distributions of synaptic weights in our model.

A large number of distinct learning rules that capture quantitatively both spike-timing and firing rate effects have been proposed recently [25]–[31]. Our rule can be distinguished from most of those rules by the fact that it includes calcium concentration as its primary dynamic variable, which allows us to extrapolate parameters of the rule from *in vitro* to *in vivo* conditions, as we have explained here. Scaling laws derived here can be expected to hold also in those other models: at low rates, the time scales of memory decay are expected to be inversely proportional to the rates to a power equal to the number of spikes needed to provoke plasticity. This power should be equal to 2 for standard STDP rules, triplet rules [25], and calcium-based rules in which 2 spikes are needed to cross the depression threshold [24], [27]; 1 for spike and voltage based rules [26].

In this work, we have made the hypothesis that synaptic weights are altered during background activity, and that one can treat background activity as being essentially uncorrelated with the synaptic connectivity structure. Memory time scales could in principle be further extended by two factors. A first mechanism would be to gate plasticity by specific neuromodulator(s) that are present only during stimulus presentation. This idea is consistent with a growing body of experimental data showing how plasticity is modulated by dopamine [63], acetylcholine [64], [65], noradrenaline [66] (see also [67] and references therein). However, we note that the model we have used here is built from *in vitro* plasticity data where these neuromodulators were present at very low concentrations, if at all. Hence, we believe that these neuromodulators are likely to *enhance* plasticity during behaviourally relevant epochs, but that the memory time scales discussed here are likely not to be affected if neuromodulators are not present at high levels during background activity.

A second mechanism that would extend memory time scales would be a scenario in which background activity is in fact strongly correlated with the connectivity structure, and wanders stochastically between network states that are strongly correlated with the states of the network during stimuli presentation. This idea is consistent with a growing experimental literature [68]–[70] showing how spontaneous activity is transiently strongly correlated with sensory responses in visual and auditory cortices, and it is also consistent with the ubiquitous supra-Poissonian variability, potentially due to the doubly-stochastic process of combined rate stochasticity and individual neuronal Poisson spike processes, seen in background activity in cortex [13], [71]. Recurrence of activity states resembling the network activity during stimulus presentation could refresh existing memory traces and therefore prolong their lifetimes.

We showed here that the low extracellular calcium concentrations *in vivo* could have a strong impact on plasticity. A first prediction of calcium-based rules is that plasticity seen in standard protocols should be greatly reduced (and even possibly vanish altogether) at physiological calcium concentrations. While to our knowledge no study has explicitly compared plasticity results at different extracellular calcium concentration, comparisons between different studies using different extracellular concentrations seem to be consistent with this prediction. In hippocampal slices, a standard low frequency STDP protocol produces LTD for all time differences with 2 mM extracellular calcium [9], while it produces the standard STDP curve with 3 mM calcium [10]. A second prediction is that induced synaptic changes should be much more stable in the face of ongoing pre- and postsynaptic activity. These results emphasise the need for experimental studies at physiological calcium concentrations 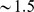 mM [72], unlike most published studies that used concentrations in the range 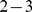 mM. Our predictions could be easily tested in slice experiments, by providing background activity at a specified rate after the plasticity-inducing protocol. Similar experiments have been performed in the developing Xenopus retino-tectal system in vivo [73], where activity-induced modifications were shown to be erased by subsequent 10 minutes of spontaneous activity. Our model would predict that in cortical slices, at 2.5 mM calcium, induced synaptic changes should disappear on a time scale of minutes, while at 1.5 mM calcium, they should be stable on a time scale of 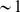 hour.

We provided here an event-based update scheme of plastic synapses which greatly accelerates simulations and should strongly facilitate future studies of the dynamics of recurrent networks with plastic calcium-based synapses. On the theoretical front, it would be interesting to extend the theory to non-Poissonian renewal processes [74] such as for leaky integrate-and-fire neurons used here, which would give a better approximation of average synaptic efficacies, especially at higher firing rates. It would also be of great interest to examine how synaptic connectivity is modulated by non-stationary external inputs, and how such changes in connectivity affect in turn the intrinsic dynamics of the network.

Our investigations show that realistic external calcium concentration and multi-stability of synapses might stabilise memory traces against the potentially deleterious effect of ongoing background activity. These results call for studies of synaptic plasticity induction and maintenance in more realistic conditions and ideally in the intact animal. They provide a glimpse of how plasticity results obtained *in vitro* might translate to the living organism.

## Materials and Methods

### Calcium-based plasticity model

The temporal dynamics of the synaptic efficacy in the calcium-based model are given in Eq. (1) (for details see [28]).

Changes in 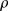 are driven by the postsynaptic calcium concentration, *c*. The calcium dynamics are modelled using instantaneous increases of size 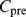 and 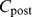 in response to pre- and postsynaptic spikes, respectively, followed by an exponential decay 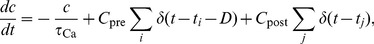
(11)where 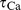 is the calcium decay time constant, and 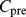, 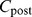 the pre- and postsynaptically evoked calcium amplitudes. The sums go over all pre- and postsynaptic spikes occurring at times 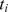 and 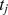, respectively. The time delay, *D*, between the presynaptic spike and the occurrence of the corresponding calcium transient accounts for the slow rise time of the NMDAR-mediated calcium influx.

We use two parameter sets in this paper. The *in vitro* parameter set is obtained by fitting the calcium-based plasticity model to plasticity data obtained in cortical slices ([6]; see [28] for details of the fitting procedure). These experiments were performed with 2.5 mM extracellular calcium concentration. The *in vivo* calcium amplitudes are obtained by scaling 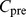 and 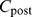 according to the extracellular calcium concentration *in vivo*, estimated to be 1.5 mM [41] (see Results).

### Probability density function of the calcium concentration

We shortly describe here how the probability density function (PDF) of the calcium concentration can be calculated if pre- and postsynaptic neurons fire as independent Poisson processes at rate 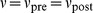 (see [28], [43] for more details). In these conditions, the calcium concentration is a shot noise process, whose probability density function is given by the master equation [43], 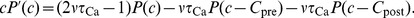
(12)


The probability density function 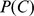 allows us to calculate the fraction of time spent above the depression and potentiation thresholds according to 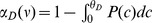 and 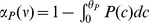.

In the simple case 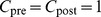, the solution to Eq. (12) is given by 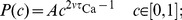
(13)

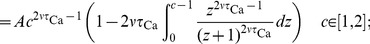
(14)

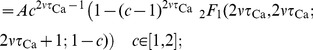
(15)

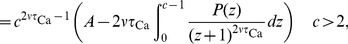
(16)where 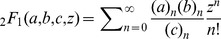 is the ordinary hypergeometric function [75], 
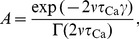
(17)

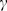
 is Euler-Mascheroni constant, 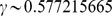, and 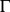 is the gamma function.

Fitting the calcium-based model to cortical plasticity data yields 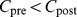 (see Table 1). In this case, the solution of Eq. (12) reads 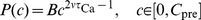
(18)

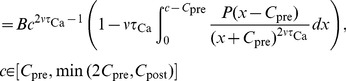
(19)

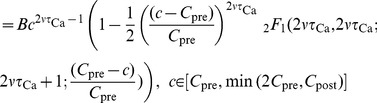
(20)where 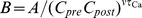 is a normalisation parameter which assures that 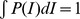. The PDF for 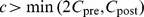 is obtained from a numerical integration of Eq. (12).

### Diffusion approximation for the synaptic efficacy with a flat potential

Performing a diffusion approximation of the synaptic efficacy 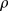 turns Eq. (1) into an Ornstein-Uhlenbeck process (see [28] for details). The temporal evolution of 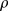 is then described by 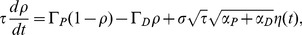
(21)for the case of a flat potential (*i.e.* 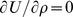). 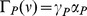 and 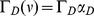 are the mean potentiation and depression rates, respectively.

The bounds at 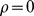 and 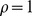 lead to a truncated Ornstein-Uhlenbeck process, whose distribution is a truncated Gaussian, whose mean converges exponentially to 
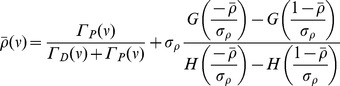
(22)where 
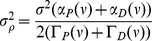
, 
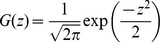
 is the Gaussian with zero mean and unit variance, and 
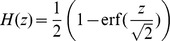
 is the complementary cumulative density function of *G*. The time constant, 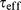, of the exponential decay to 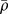 is defined in Eq. (3).

### Kramers expected escape time from a double-well potential

In the case of a double-well potential, the diffusion approximation turns Eq. (1) into a Fokker-Planck equation 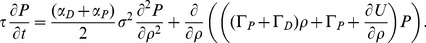
(23)


This equation can be rewritten as 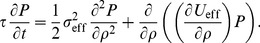
(24)where the *effective potential*, 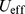, is the sum of the ‘bare’ potential *U* and two quadratic terms proportional to the potentiation and depression rates, respectively (see Eq. (10)), and 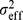 is the amplitude of the effective noise



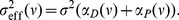
(25)When the effective potential is dominated by the double-well term (first term on the rhs of Eq. (10)), the ‘escape’ rate from the UP state is driven by noise and can be estimated using Kramers theory [76], [77]. The height of the potential barrier, 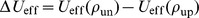, determines the mean dwell time in the UP state, where 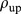 and 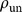 are the local minima and maxima of the effective potential and are obtained solving 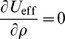. This allows us to calculate the expected escape time from the potential well 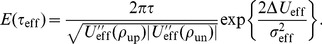
(26)


### Numerical methods: Event-based implementation

The temporal evolution of individual synaptic weights in the calcium-based model can be calculated in an event-based manner (as opposed to a finite difference method with a fixed time step 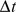) in an analytically exact way. This greatly accelerates network simulations since the network variables are updated at the occurrence of spikes only. In the following we describe the practical implementation of the event-based update.

For the event-based update, three variables have to be stored per synapse: the calcium concentration, *c*, the synaptic efficacy variable, 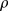, and the time of the last spike seen by the synapse, *t*. The synapse variables *c* and 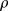 must be updated on the occurrence of three events: (1) at the presynaptic spike when the postsynaptic voltage is increased, (2) with delay *D* after a presynaptic spike when the presynaptically evoked calcium increase occurs (see Eq. (11)), (3) and at the postsynaptic spike when the postsynaptic calcium increase occurs.

#### Calcium update

The calcium concentration decays exponentially between events and is instantaneously increased by the amplitude 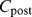 when a postsynaptic spike occurs. In the case of a presynaptic spike, the calcium increase 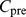 occurs after the delay *D* (Eq. (11)). In consequence, we update the calcium concentration as a decay followed by a calcium concentration increase where the amplitude depends on the identity of the spike and the delay *D* (at time 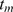 for pre-synaptic spikes and 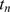 for post-synaptic spikes). The calcium update for time 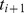 (after the last update at 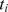) at the three update events described above reads 
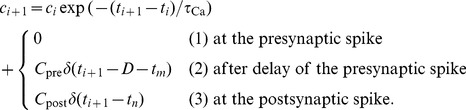
(27)


#### Synaptic efficacy update

For the propagation of the synaptic efficacy variable, we distinguish between two different regimes, that is, stochastic and deterministic. When the calcium concentration at time 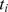 is lower than both thresholds, 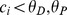, the dynamics of 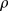 are described deterministically. When the calcium concentration crosses either or both thresholds, the update is stochastic, for the duration of the suprathreshold period, and the dynamics of the mean and the standard deviation of the synaptic efficacy are described by the corresponding Ornstein-Uhlenbeck process. The different regimes may be updated sequentially in a piecewise manner, accounting for first suprathreshold and then subthreshold behaviour.

Here, we determine the possible potentiation/depression threshold crossings of the calcium trace between events *i* and 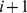 with the inter-event interval 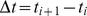. 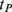 is the time the calcium trace spends above the potentiation threshold within that interval, 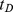 is the time the calcium trace spends between the potentiation and the depression threshold, and 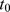 the time the calcium trace spends below the depression threshold given by 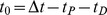. Here, we consider 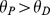 only. The updates for the case 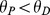 are equivalent. 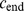 refers to the calcium concentration right before the update at event 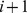, that is 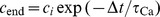.

**Figure 7:**
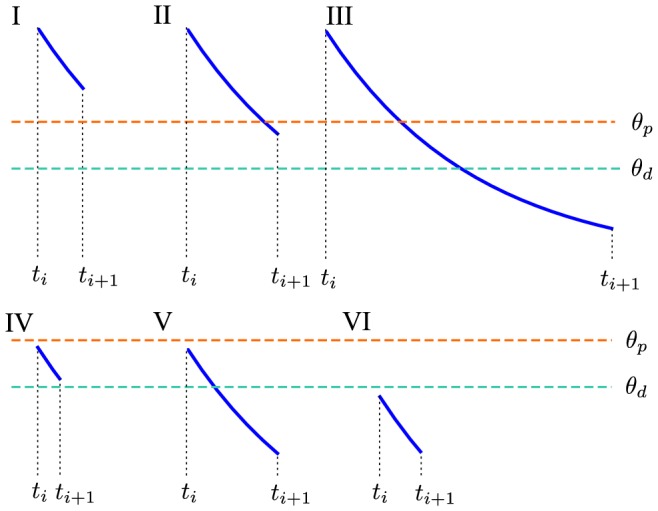
Possible potentiation and depression threshold crossing cases of the calcium trace (blue lines) between events at time 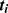 and 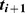. The six possible cases are depicted with respect to the location of the potentiation, 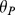 (orange dashed line), and the depression thresholds, 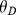 (cyan dashed line).

Between events at 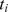 and 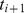, the following six possible threshold crossings can occur (see Fig. 7):
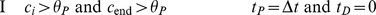


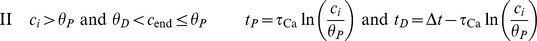


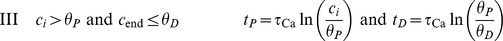


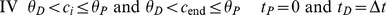


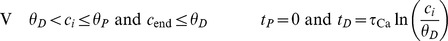


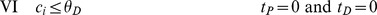



The efficacy, 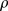, is updated in a piece-wise fashion. Stochastic updates are performed when the calcium trace spent time above the potentiation threshold (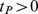, cases: I, II, III), or between the potentiation threshold and the depression threshold (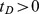, cases: II, III, IV, V; and for 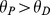). A deterministic update is performed if the calcium trace spent time below the depression threshold (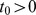, cases: III, V, VI).

In case one or both thresholds are crossed in the interval (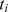,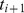], the temporal evolution of 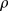 cannot be solved analytically in the double well potential case, because of the combined presence of the stochastic term and the quartic potential. However, for the parameters used in this paper the double-well potential can be neglected in the suprathreshold regions because 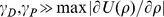 (see Table 1). We can therefore approximate the temporal evolution of 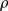 by an Ornstein-Uhlenbeck (OU) process, for which the potential of 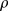 during stimulation is quadratic with the minimum at 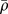. We confirmed the validity of this approximation by comparing the event-based simulation based on this approximation, with a simulation which was event-based only in sub-threshold epochs, and based on a forward Euler method in the supra-threshold epochs. The results of both simulations were indistinguishable, which confirms that we can ignore the potential well during the suprathreshold period.

Using this approximations, the updates of the synaptic variable after suprathreshold epochs are given by:
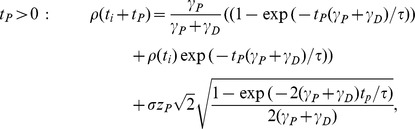


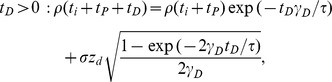
where 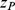 and 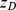 are Gaussian random variables of unit variance and zero mean. Note that 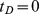 in case I, and therefore only the first update is performed. Equivalently, 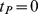 in cases IV as well as V and only the second update rule is performed.

When the calcium concentration, 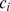, is smaller than the potentiation and the depression threshold (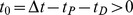, cases III, V and VI), the first two terms on the rhs and the noise term of Eq. (1) are zero. That reduces the rhs of Eq. (1) to 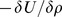 which can be integrated analytically for the flat- and the double well potential considered here. The update of 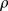 is therefore deterministic and depends on the choice of the potential:

**1. flat potential **

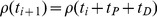
(28)


**2. double-well potential **

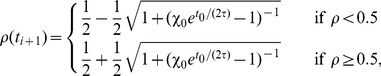
(29)with 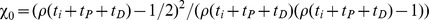.

### The network

We implemented and simulated a recurrent network of 10,000 leaky integrate-and-fire (LIF) neurons, 8,000 of which are excitatory (E) neurons and 2,000 of which are inhibitory (I). Any two neurons have a spatially uniform probability of connection of 0.05. Autapses are specifically disallowed. Synapses between E neurons are plastic and their weight dynamics are described by the calcium-based plasticity model (Eq. 1, [28]). All other synapses have fixed strength 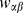 (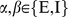). A presynaptic spike induces a voltage jump of size 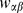 in the postsynaptic neuron.

The membrane potential of neuron *i* of population 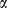 evolve according to 
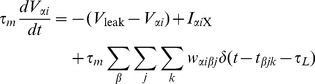
(30)where
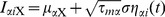
(31)is a common external drive to all neurons, comprising a constant input, 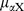, and a white noise of amplitude 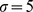
mV. 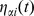 is a Gaussian white noise process with unit variance and zero mean, which is uncorrelated from neuron to neuron. In the absence of synaptic inputs each membrane potential decays exponentially to the leak potential, 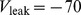 mV, with a time constant 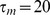 ms. Spiking occurs when the voltage crosses a threshold, 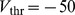 mV, after which it is reset to the reset potential, 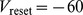 mV. During all of our simulations, we set the refractory period, during which the membrane potential is fixed at 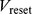 after spiking, to zero. The three sums in the r.h.s. of Eq. (30) go over the two populations 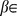
{E, I}, all presynaptic neurons *j*, and presynaptic spikes of neuron *j* in population 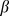, that occur at times 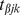. Each presynaptic spike of neuron *j* in population 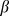 causes a jump of amplitude 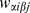 in the voltage of neuron *i* after a delay 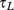. Here, the delay is chosen to be equal to the integration time step 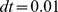 ms (see below).

**Figure 8:**
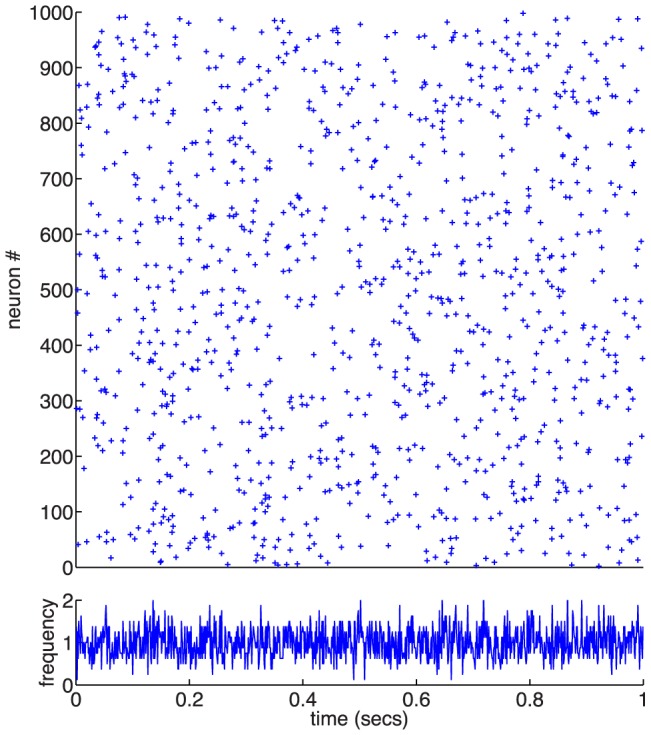
Example of network firing in asynchronous irregular state. A sample of 1000 neurons from the network shows irregular spiking behaviour in the raster (top) and the averaged firing rate of all 8000 excitatory neurons is steady artabound 1/sec (bottom).

For all connections involving inhibition (i.e. all 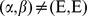), the connectivity matrix is set as 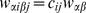 where 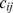 are independent, identically distributed (i.i.d.) Bernoulli variables, 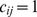 with probability 0.05, 0 with probability 0.95, and the fixed synaptic weights are 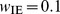
mV, 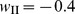
mV and 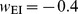
mV. E-E synapses are given by 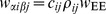 where 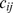 are again i.i.d. Bernoulli variables, 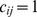 with probability 0.05, 0 with probability 0.95, 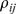 obeys Eq. (1), and 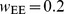
mV. The average value of 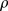 is initially, and remains throughout our simulations, much smaller than 0.5, which means that with a 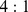 ratio in the E to I populations, for 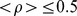 recurrent inhibition dominates excitation, leading to a stable asynchronous irregular state (see Fig. 8) [44].

### Numerical methods: Network simulations

We numerically simulated the recurrent network of LIF neurons using the forward Euler method with a time step of 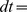
0.01 ms. Synapses were updated using the event-based implementation described above. The simulations were implemented in C and OpenCL and run on general-purpose GPUs. Parallel generation of random numbers, for the Gaussian noise in the LIF equations, was implemented using the Random123 library [78].

In order to initialise the simulations close to their steady-state, with the *in-vivo* parameter set and the double-well potential, we first calculate the probability distribution function (PDF) for the synaptic weights assuming a 1/s pre- and post-synaptic Poisson firing process. We then use a reverse lookup of the associated cumulative distribution function (CDF) to determine the random initial values for the synaptic efficacies.

### Computing analytically mean firing rates and E-E synaptic efficacy

In a network of excitatory and inhibitory LIF neurons receiving white noise inputs, the mean firing rates of excitatory and inhibitory neurons are given by [44], [45]

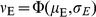
(32)

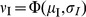
(33)where 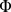 is the standard LIF static transfer function [44], [45], [79], [80],
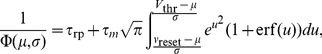
(34)where 
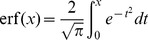
 is the error function, 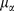 are the mean inputs to population 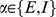,



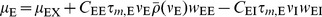
(35)

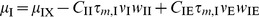
(36)and 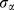 is the amplitude of the fluctuations in the inputs to population 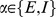,



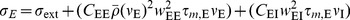
(37)

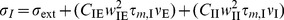
(38)


Note that in Eqs. (35,37) 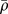 is given by Eq. (22). Note also that for the parameters studied in this paper the effect of heterogeneities in numbers of inputs [81], [82] have a negligible effect on the mean firing rates of the network.
